# Crucial Role of NLRP3 Inflammasome in the Development of Peritoneal Dialysis-related Peritoneal Fibrosis

**DOI:** 10.1038/s41598-019-46504-1

**Published:** 2019-07-17

**Authors:** Erika Hishida, Homare Ito, Takanori Komada, Tadayoshi Karasawa, Hiroaki Kimura, Sachiko Watanabe, Ryo Kamata, Emi Aizawa, Tadashi Kasahara, Yoshiyuki Morishita, Tetsu Akimoto, Daisuke Nagata, Masafumi Takahashi

**Affiliations:** 1 Division of Inflammation Research, Center for Molecular Medicine, Jichi Medical University, Tochigi, Japan; 2 Division of Nephrology, Department of Internal Medicine, Jichi Medical University, Tochigi, Japan; 3 Division of Nephrology, Department of Integrated Medicine, Saitama Medical Center, Jichi Medical University, Saitama, Japan

**Keywords:** Peritoneal dialysis, Kidney

## Abstract

Long-term peritoneal dialysis (PD) therapy leads to peritoneal inflammation and fibrosis. However, the mechanism underlying PD-related peritoneal inflammation and fibrosis remains unclear. NLRP3 inflammasome regulates the caspase-1-dependent release of interleukin-1β and mediates inflammation in various diseases. Here, we investigated the role of NLRP3 inflammasome in a murine model of PD-related peritoneal fibrosis induced by methylglyoxal (MGO). Inflammasome-related proteins were upregulated in the peritoneum of MGO-treated mice. MGO induced parietal and visceral peritoneal fibrosis in wild-type mice, which was significantly reduced in mice deficient in NLRP3, ASC, and interleukin-1β (IL-1β). ASC deficiency reduced the expression of inflammatory cytokines and fibrotic factors, and the infiltration of macrophages. However, myeloid cell-specific ASC deficiency failed to inhibit MGO-induced peritoneal fibrosis. MGO caused hemorrhagic ascites, fibrin deposition, and plasminogen activator inhibitor-1 upregulation, but all of these manifestations were inhibited by ASC deficiency. Furthermore, *in vitro* experiments showed that MGO induced cell death via the generation of reactive oxygen species in vascular endothelial cells, which was inhibited by ASC deficiency. Our results showed that endothelial NLRP3 inflammasome contributes to PD-related peritoneal inflammation and fibrosis, and provide new insights into the mechanisms underlying the pathogenesis of this disorder.

## Introduction

Peritoneal dialysis (PD) is a renal replacement therapy used by approximately 200,000 patients with end-stage kidney disease worldwide, accounting for 11% of the PD population. Long-term PD leads to fibrotic changes of the peritoneum with functional decline^1,2^. In addition, peritoneal fibrosis can, albeit rarely, progresses to fibrous encapsulation of the small bowels as a “cocoon”, known as encapsulating peritoneal sclerosis (EPS), which is associated with high morbidity and mortality. The pathological feature of EPS is fibrin deposition due to plasma exudation from peritoneal microvessels^3,4^. Recent studies have demonstrated that inflammation induced by bio-incompatible PD fluids (PDFs) and/or infections is involved in the pathogenesis of PD-related peritoneal fibrosis^4,5^. In particular, heat sterilization of conventional PDFs leads to the generation of glucose degradation products (GDPs), such as acetaldehyde, formaldehyde, glyoxal, and methylglyoxal (MGO)^6^. Of these, MGO is an extremely toxic GDP that causes oxidative stress and peritoneal injury. MGO has also been shown to enhance the production of vascular endothelial growth factor (VEGF), which may lead to vascular permeability and procoagulant activity. Furthermore, MGO administration induces peritoneal fibrosis in experimental rodents, which is frequently used as an animal model of PD-related peritoneal fibrosis^7,8^. However, the mechanisms underlying inflammation in the development of PD-related peritoneal fibrosis have not been fully understood.

Accumulating body of evidence suggests that inflammation in the absence of pathogens (sterile inflammation) is mediated via the inflammasome; an intracellular multi-protein complex that regulates release of proinflammatory cytokine interleukin (IL)-1β^9–11^. Although several pattern recognition receptors (PRRs) can form the inflammasome complex, the nucleotide-binding oligomerization domain-like receptor (NLR) family pyrin domain containing 3 (NLRP3) inflammasome is well characterized and has been implicated in sterile inflammatory diseases. The NLRP3 inflammasome contains NLRP3 associated with an apoptosis-associated speck-like protein containing a caspase recruitment domain (ASC), which recruits caspase-1 and induces its activation. Since caspase-1 is an IL-1β-converting enzyme^12^, NLRP3 inflammasome-driven caspase-1 activation induces the processing of pro-IL-1β into its mature form, and the release of this potent inflammatory cytokine IL-1β causes tissue inflammation and damage. Furthermore, activation of NLRP3 inflammasome also triggers inflammatory cell death termed as pyroptosis^9–11^. Our group recently demonstrated that NLRP3 inflammasome is involved in sterile inflammatory responses in cardiovascular and renal diseases^13–17^. Supporting our findings, recent investigations have also shown that NLRP3 inflammasome is a key mediator of many sterile inflammation-related diseases including gout, atherosclerosis, type 2 diabetes, and metabolic syndrome^18^. We and other investigators have also shown that NLRP3 inflammasome is involved in the expression of fibrotic factors and the development of tissue fibrosis^15,17,19^. Additionally, NLRP3 itself has been suggested to contribute to the signaling of transforming growth factor-β (TGF-β) in tubular epithelial cells and to promote renal fibrosis independent of the inflammasome^20^. Further, Hautem *et al*.^21^ recently reported that NLRP3 inflammasome plays a role in transport defects and morphological alterations in PD-related bacterial peritonitis. However, the role of NLRP3 inflammasome in PD-related peritoneal fibrosis remains to be elucidated.

In the present study, we used mice deficient in NLRP3, ASC, and IL-1β, and generated MGO-induced peritoneal fibrosis, which is an excellent murine model for human PD-related peritoneal fibrosis. We clearly showed that deficiency of NLRP3, ASC, or IL-1β reduced inflammatory and fibrotic responses in a MGO-induced peritoneal fibrosis model. MGO also caused peritoneal bleeding and fibrin deposition, and subsequently promoted peritoneal fibrosis, all of these manifestations were inhibited by ASC deficiency. *In vitro* experiments showed that MGO generated reactive oxygen species (ROS) and induced cell death in endothelial cells, which was inhibited by ASC deficiency. The present findings showed that endothelial NLRP3 inflammasome contributes to peritoneal inflammatory and fibrotic responses in the pathophysiology of PD-related peritoneal fibrosis, and provide new insights into the mechanisms underlying the pathogenesis of this disorder.

## Results

### Inflammasome-related proteins were up-regulated in MGO-induced peritoneal fibrosis

**Figure 1:**
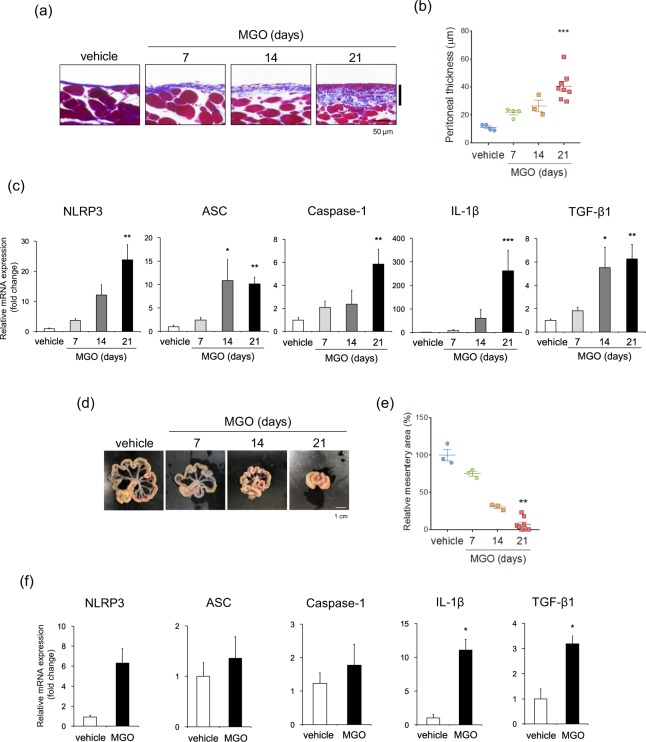
Inflammasome-related molecules were up-regulated in MGO-induced peritoneal fibrosis. WT mice were injected with vehicle or MGO for the indicated periods. (**a**) Representative images of MT staining in parietal peritoneum. Black bars indicate the parietal peritoneal thickness (degree of fibrosis). (**b**) Quantitative analysis of peritoneal thickness (n = 3–8 for each). (**c**) mRNA expression of NLRP3, ASC, caspase-1, IL-1β, and TGF-β in the parietal peritoneum was assessed by using real-time RT-PCR analysis (n = 3–8 for each). (**d**) Representative images of mesentery. (**e**) Quantitative analysis of the mesentery area (n = 3–8 for each). (**f**) mRNA expression of NLRP3, ASC, caspase-1, IL-1β, and TGF-β in the visceral peritoneum was assessed (day 14, n = 3 for each). Data are expressed as means ± SEM. *p < 0.05, **p < 0.01, ***p < 0.001.

To produce a murine model of MGO-induced peritoneal fibrosis, we injected MGO (40 mM) intraperitoneally into wild-type (WT) mice, and Masson’s Trichrome (MT) staining showed that MGO significantly induced fibrous thickening of the parietal peritoneum in a time-dependent manner (Fig. 1a,b). We next assessed the expression of inflammasome-related proteins by using real-time RT-PCR analysis and found that the peritoneal expression of NLRP3, ASC, caspase-1, and IL-1β was elevated in a time-dependent manner, and this increase was statistically significant at 21 days after MGO treatment (Fig. 1c). We also assessed the expression of other inflammasome-forming PRRs, there was no significant differences of the mRNA expression of NLRP1, NLRC4 (NLR family CARD-containing 4), and AIM2 (absent in melanoma 2) between vehicle- and MGO-treated groups (Supplementary Fig. S1). The peritoneal expression of transforming growth factor (TGF)-β was also significantly elevated at 14 and 21 days after MGO treatment. To further investigate the peritoneal fibrotic process, we assessed fibrotic changes in the visceral peritoneum and found that MGO induced shrinkage of the mesentery, and its area was apparently decreased after MGO treatment in a time-dependent manner (Fig. 1d,e). Furthermore, some mice exhibited bowel adhesions, a clinical presentation similar to EPS, at 21 days after MGO treatment. Consistently, the expression of inflammasome-related molecules tended to be elevated in the visceral peritoneum after MGO treatment (Fig. 1f).

### Deficiency of NLRP3 or ASC reduced MGO-induced peritoneal fibrosis

**Figure 2:**
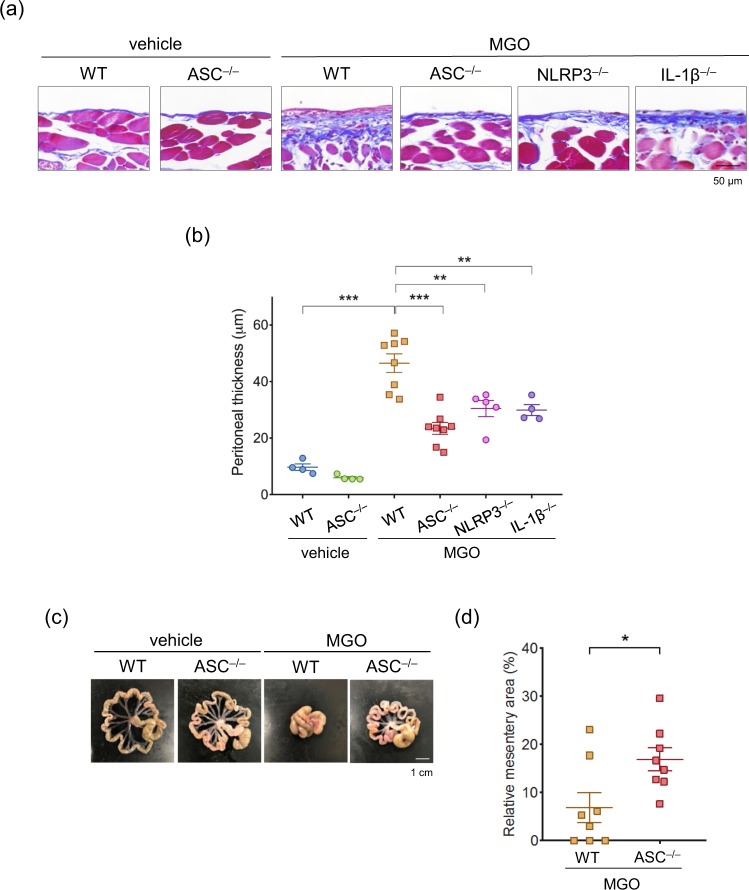
Deficiency of NLRP3 and ASC reduced MGO-induced peritoneal fibrosis. WT, ASC^–/–^, NLRP3^–/–^, and IL-1β^–/–^ mice were injected with vehicle or MGO for 21 days. (**a**) Representative images of MT staining in parietal peritoneum. (**b**) Quantitative analysis of peritoneal thickness (n = 3–8 for each). (**c**) Representative images of mesentery. (**d**) Quantitative analysis of the mesentery area (n = 8 for each). Data are expressed as means ± SEM. *p < 0.05, **p < 0.01.

To investigate the role of NLRP3 inflammasome, we used NLRP3^–/–^, ASC^–/–^, and IL-1β^–/–^ mice, and found that a deficiency of NLRP3, ASC, or IL-1β partially but significantly reduced MGO-induced fibrosis in the parietal peritoneum (Fig. 2a,b). Because this inhibitory effect was predominantly observed in ASC^–/–^ mice, the following experiments were conducted to assess the phenotypes in WT and ASC^–/–^ mice. Consistent with the findings in the parietal peritoneum, the mesentery area was retained in ASC^–/–^ mice, compared to that in WT mice (Fig. 2c,d).

### ASC deficiency reduced inflammatory and fibrotic responses

**Figure 3:**
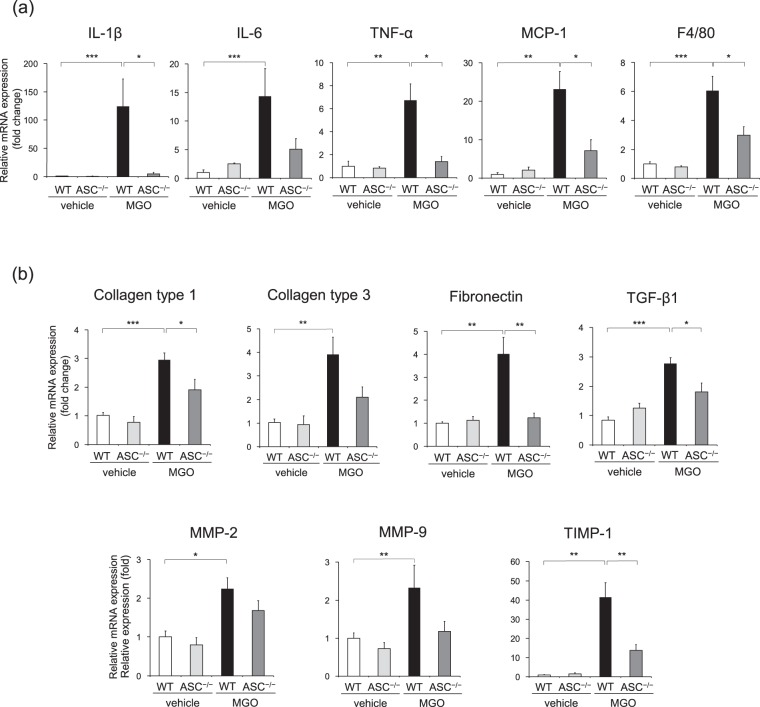
ASC deficiency reduced inflammatory and fibrotic responses. WT and ASC^–/–^ mice were injected with vehicle or MGO for 21 days. (**a**) mRNA expression of IL-1β, IL-6, TNF-α, MCP-1, and Emr1 in the parietal peritoneum was assessed by using real-time RT-PCR analysis (n = 3–8 for each). (**b**) mRNA expression of collagen type 1 and 3, fibronectin, TGF-β, MMP-2, MMP-9, and TIMP-1 in the parietal peritoneum was assessed (n = 3–8 for each). Data are expressed as means ± SEM. *p < 0.05, **p < 0.01, ***p < 0.001.

Because inflammatory response is linked to the NLRP3 inflammasome as well as PD-related peritoneal fibrosis^5,22^, we assessed the expression of inflammatory molecules in the parietal peritoneum, and showed that the mRNA expression of IL-1β, IL-6, tumor necrosis factor (TNF)-α, monocyte chemoattractant protein (MCP)-1, and F4/80 (a macrophage marker; Emr1) was markedly increased in the MGO-treated peritoneum of WT mice, but the increased expression of these cytokines and macrophage marker was decreased in ASC^–/–^ mice (Fig. 3a). As expected from the data on fibrosis, similar expression patterns were observed in the expression of collagen type 1 and type 3, fibronectin, TGF-β, matrix metalloproteinase (MMP)-2, MMP-9, and tissue inhibitor of metalloproteinase (TIMP)-1 (Fig. 3b). Analysis of the visceral peritoneum also showed similar changes in the expression of inflammatory and fibrotic factors (Supplementary Fig. S2).

### ASC deficiency reduced the inflammatory cell infiltration

**Figure 4:**
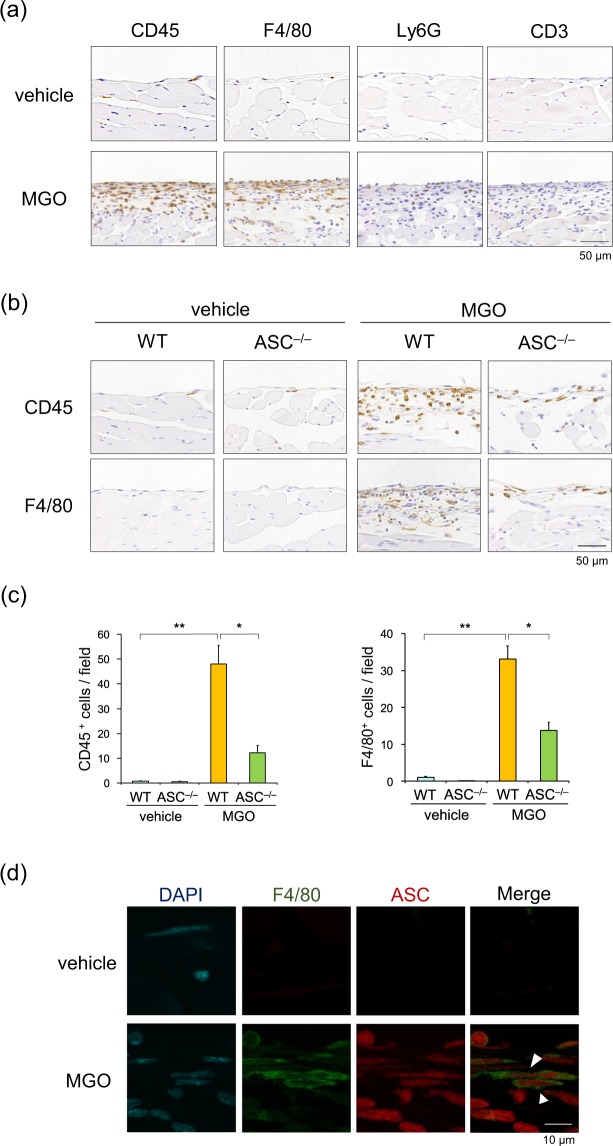
ASC deficiency reduced the inflammatory cell infiltration. WT and ASC^–/–^ mice were injected with vehicle or MGO for 21 days. (**a**) Sections of the parietal peritoneum in WT mice were immunohistochemically stained with antibodies against CD45, F4/80, Ly6G, and CD3. (**b**) Sections of the parietal peritoneum in WT and ASC^–/–^ mice were immunohistochemically stained with antibodies against CD45 and F4/80. (**c**) Quantitative analysis of CD45- and F4/80-positive cells (n = 3-4 for each). (**d**) Sections of the parietal peritoneum in WT mice were analyzed by double-immunofluorescence staining with antibodies against F4/80 (green) and ASC (red). DAPI (blue) indicates nuclear staining. Data are expressed as means ± SEM. *p < 0.05, **p < 0.01.

Since the components of NLRP3 inflammasome are expressed mainly in inflammatory cells, especially macrophages^10,15^, we performed an immunohistochemical analysis to analyze infiltrated inflammatory cells. The infiltration of CD45^+^ (pan-leukocyte marker) and F4/80^+^ (macrophage marker) cells, but not Ly6G^+^ (neutrophil marker) or CD3^+^ (T cell marker) cells, was markedly increased in the parietal peritoneum of MGO-treated WT mice (Fig. 4a). The infiltration of CD45^+^ and F4/80^+^ cells was significantly inhibited in ASC^–/–^ mice (Fig. 4b,c). Furthermore, double immunofluorescence staining revealed the colocalization of ASC with F4/80^+^ cells (Fig. 4d).

### Myeloid cell-specific ASC deficiency had no effect on MGO-induced peritoneal fibrosis

**Figure 5:**
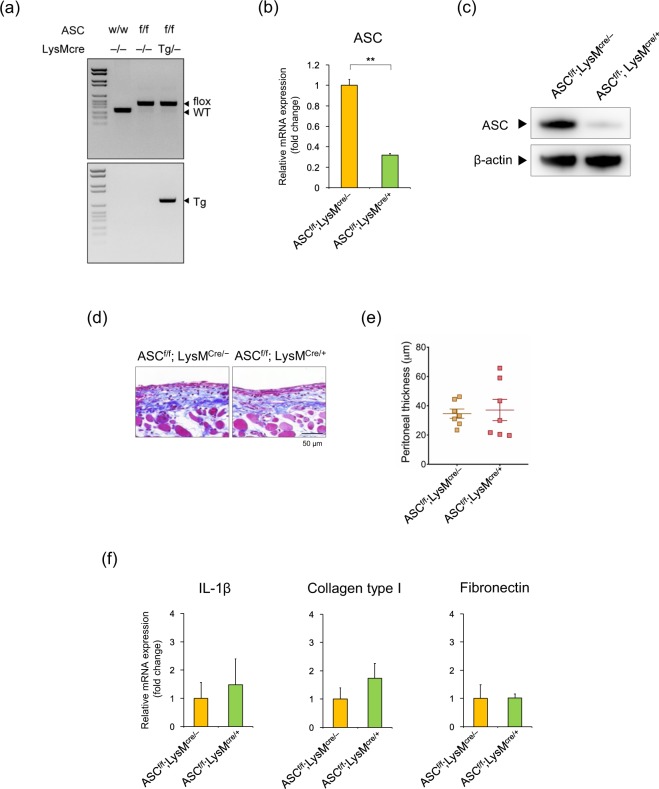
Myeloid cell-specific ASC-KO mice had no effect on MGO-induced peritoneal fibrosis. (**a**) Genotyping of ASC^f/f^;LysM^cre/–^ and ASC^f/f^;LysM^cre/+^ mice. Floxed ASC gene and presence of Cre gene in genome DNA were confirmed by PCR analysis. (**b**,**c**) ASC mRNA (**b**) and protein (**c**) levels in bone marrow-derived macrophages from ASC^f/f^;LysM^cre/–^ and ASC^f/f^;LysM^cre/+^ mice were analyzed by real-time RT-PCR and western blot analyses, respectively. β-actin served as a loading control. (**d**–**f**) ASC^f/f^;LysM^cre/–^ and ASC^f/f^;LysM^cre/+^ mice were injected with vehicle or MGO for 21 days. (**d**) Representative images of MT staining in the parietal peritoneum. (**e**) Quantitative analysis of peritoneal thickness (n = 7 for each). (**f**) mRNA expression of IL-1β, collagen type 1, and fibronectin in the parietal peritoneum was assessed by using real-time RT-PCR analysis (n = 4 for each). Data are expressed as means ± SEM (n = 4). *p < 0.05, **p < 0.01.

To determine the contribution of the macrophage NLRP3 inflammasome, we used two different strategies: bone marrow transplantation (BMT) and myeloid cell-specific ASC^–/–^ mice. First, we generated 3 types of BMT mice (BMT^WT to WT^, BMT ^ASC–/– to WT^, BMT^WT to ASC–/–^) and produced MGO-induced peritoneal fibrosis in these mice. Unfortunately, MGO treatment caused chylous ascites in these BMT mice (Supplementary Fig. S3A,B). Furthermore, thickness of the parietal peritoneum did not differ between these groups (Supplementary Fig. S3C,D). Second, we generated myeloid cell-specific ASC^–/–^ mice (ASC^f/f^;LysM^cre/+^) using the Cre-loxP gene-targeting technique (Fig. 5a, Supplementary Fig. S4). The expression of ASC mRNA and protein was markedly decreased in bone marrow-derived macrophages (BMDMs) isolated from ASC^f/f^;LysM^cre/+^ mice, compared to those from ASC^f/f^;LysM^cre/–^ mice (Fig. 5b,c). In addition, IL-1β production in response to an NLRP3 activator nigericin was also inhibited in ASC^f/f^;LysM^cre/+^ mice (Supplementary Fig. S5). Contrary to our expectations, myeloid cell-specific ASC deficiency had no significant effect on MGO-induced peritoneal fibrosis (Fig. 5d,e). Furthermore, the expression levels of IL-1β, collagen type 1, and fibronectin in the peritoneum did not differ between ASC^f/f^;LysM^cre/–^ and ASC^f/f^;LysM^cre/+^ mice (Fig. 5f).

### ASC deficiency exhibited less peritoneal bleeding and fibrin deposition

**Figure 6:**
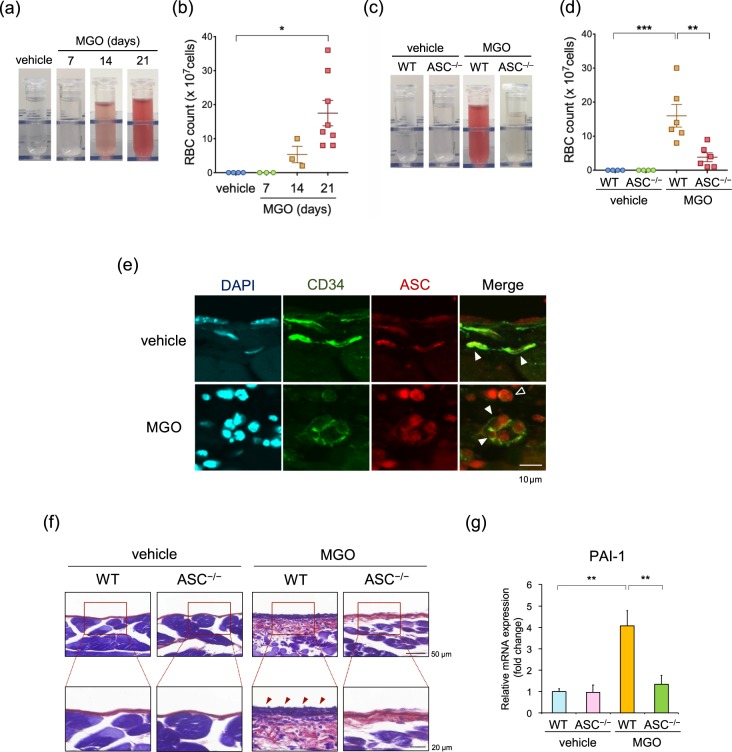
ASC deficiency was associated with less peritoneal bleeding and fibrin deposition. (**a–d**) WT and ASC^–/–^ mice were injected with vehicle or MGO for the indicated periods. (**a**) Representative images of peritoneal effluents in WT mice. (**b**) Quantitative analysis of RBC count in the peritoneal effluents in WT mice (n = 3–8 for each). (**c**) Representative images of peritoneal effluents in WT and ASC^–/–^ mice (n = 4–6 for each). (**d**) Quantitative analysis of RBC count in the peritoneal effluents in WT and ASC^–/–^ mice. (**e**) Sections of the parietal peritoneum in WT mice were analyzed by double-immunofluorescence staining with antibodies against CD34 (green) and ASC (red). DAPI (blue) indicates nuclear staining. Arrowheads and open arrowheads indicate endothelial cells and infiltrated macrophages, respectively. (**f**) Representative images of phosphotungstic acid-hematoxylin (PTAH) staining for fibrin deposition in the parietal peritoneum. (**g**) mRNA expression of PAI-1 in the parietal peritoneum was assessed by using real-time RT-PCR analysis (n = 3–8 for each). Data are expressed as means ± SEM. *p < 0.05, **p < 0.01, ***p < 0.001.

Previous investigations have suggested that endothelial cells play a role in the development of PD-related peritoneal fibrosis^23^. Indeed, we observed that MGO treatment caused hemorrhagic ascites in a time-dependent manner (Fig. 6a) and the red blood cell (RBC) counts in the peritoneal lavage were significantly increased at 21 days after MGO treatment (Fig. 6b). This MGO-induced hemorrhagic ascites was considerably prevented in ASC^–/–^ mice (Fig. 6c,d). To determine whether endothelial cells express ASC, we performed an immunohistochemical analysis and found that serial sections stained for CD34 (endothelial marker) revealed that ASC was expressed in endothelial cells (Fig. 6e). Fibrin deposition was also increased in the MGO-treated peritoneum of WT mice, and this deposition was markedly decreased in the peritoneum of ASC^–/–^ mice (Fig. 6f). Furthermore, the expression of plasminogen activator inhibitor -1 (PAI-1), a marker for endothelial dysfunction, was significantly increased in the MGO-treated peritoneum of WT mice, whereas this increase was inhibited in ASC^–/–^ mice (Fig. 6g).

### Roles of endothelial ROS generation, cell death, and IL-1β

**Figure 7:**
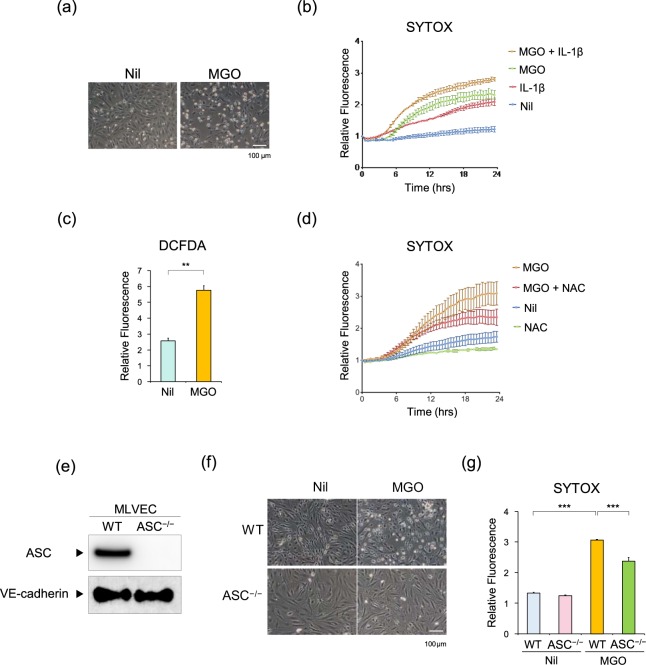
Roles of endothelial ROS generation, cell death, and IL-1β. (**a**) Representative images of HUVECs treated with or without 1 mM MGO for 24 h. (**b**) HUVECs were treated with MGO (1 mM) in the presence or absence of human IL-1β (100 ng/mL). Cell death was detected with SYTOX Green and the fluorescence intensity was measured at 30-min intervals (n = 4 for each). (**c**) HUVECs were treated with MGO (1 mM) for 3 h. ROS was detected with DCFDA and the fluorescence intensity was measured (n = 4 for each). (**d**) HUVECs were treated with MGO (1 mM) in the presence or absence of NAC (25 mM). Cell death was detected with SYTOX Green and the fluorescence intensity was measured at 30-min intervals (n = 4 for each). (**e**) The expression levels of ASC and VE-cadherin in MLVECs from WT and ASC^−/−^ mice were analyzed by western blot analysis. (f,g) WT and ASC^−/−^ MLVECs were treated with MGO (1 mM) for 12 h. (**f**) Representative images of MLVECs. (**g**) Cell death was detected with SYTOX Green and the fluorescence intensity was measured (n = 4 for each). Data are expressed as means ± SEM. **p < 0.01, ***p < 0.001.

To explore the mechanism by which ASC deficiency prevents MGO-induced peritoneal fibrosis, we used two types of cultured endothelial cells: human umbilical vein endothelial cells (HUVECs) and murine lung vascular endothelial cells (MLVECs). Because we could not observe MGO-induced IL-1β production in HUVECs and MLVECs primed with or without lipopolysaccharide (data not shown), we then focused on endothelial cell death and found that MGO (1 mM) treatment clearly induced cell death of HUVECs in a time-dependent manner (Fig. 7a,b). IL-1β treatment also induced cell death and additively enhanced MGO-induced cell death. Because MGO has been shown to generate ROS^24^, we assessed ROS levels using the 2’,7’ –dichlorofluorescein diacetate (DCFDA) method and showed that MGO significantly promoted ROS generation in HUVECs (Fig. 7c). Furthermore, MGO-induced cell death was significantly inhibited by pretreatment with the antioxidant N-acetyl cysteine (NAC) (Fig. 7d). To assess whether ASC deficiency could inhibit MGO-induced cell death, we prepared primary MLVECs (CD45^–^CD31^+^) from WT and ASC^–/–^ mice by using negative immune-magnetic selection (Fig. 7e). MGO treatment induced cell death in WT MLVECs, which was prevented by ASC^–/–^ MLVECs (Fig. 7f,g).

## Discussion

The major findings of this study are as follows: 1) Inflammasome-related proteins were upregulated in the peritoneum of MGO-treated mice; 2) MGO induced parietal and visceral peritoneal fibrosis in WT mice, and this fibrosis was significantly reduced in mice deficient in NLRP3, ASC, and IL-1β; 3) ASC deficiency reduced the expression of inflammatory cytokines and fibrotic factors, and the infiltration of macrophages; however, the myeloid cell-specific ASC deficiency failed to inhibit MGO-induced peritoneal fibrosis; 4) MGO caused hemorrhagic ascites, fibrin deposition, and the expression of PAI-1, and all of these manifestations were inhibited by ASC deficiency; and 5) *In vitro* experiments showed that MGO induced cell death via the generation of ROS in endothelial cells, which was inhibited by ASC deficiency. The present results indicate that NLRP3 inflammasome plays a crucial role in inflammatory and fibrotic responses in the development of PD-related peritoneal fibrosis, and provide new insights into the mechanism underlying the pathogenesis of PD-related peritoneal fibrosis.

Prolonged exposure to bio-incompatible PDFs causes cellular stress and tissue injury which lead to sterile peritoneal inflammation, resulting in peritoneal fibrosis and EPS^5,25^. However, the mechanisms underlying sterile peritoneal inflammation resulting from prolonged exposure to PDFs and leading to peritoneal fibrosis and EPS remain to be elucidated. Meanwhile, we and other investigators have recently shown that NLRP3 inflammasome drives sterile inflammation in various diseases, such as cardiovascular and renal diseases^9,10,18^. In the present study, we showed that deficiency of NLRP3, ASC, and IL-1β attenuates inflammatory and fibrotic responses after MGO treatment, resulting in less peritoneal fibrosis in a murine model of PD-related peritoneal fibrosis and EPS. Several murine models are commonly used to study the pathogenesis of PD-related peritoneal fibrosis including the administration of chlorhexidine gluconate (CG), acidic (pH 3.8) glucose solution, and MGO^22^. Although some histological features of PD-related peritoneal fibrosis are seen in these models, it is accepted that MGO-induced peritoneal fibrosis is more appropriate for studying the pathogenesis of PD-related peritoneal fibrosis because conventional PD fluids contain GDPs including MGO. In addition, the MGO model exhibits histological hallmarks similar to those of PD-related peritoneal fibrosis, such as thickening of the submesothelial compact zone, infiltration of inflammatory cells, capillary angiogenesis, and fibrin deposition^22^. Intriguingly, in addition to these histological features, we found that MGO treatment induces shrinkage of the mesentery and bowel adhesions, which are clinical presentations similar to those in EPS, and these are inhibited by ASC deficiency. To our knowledge, this study provides the first evidence that NLRP3 inflammasome plays a role in sterile peritoneal inflammation and subsequent fibrosis in PD-related peritoneal fibrosis and EPS.

Recent investigations have suggested that inflammatory cells, particularly macrophages, contribute to the process of peritoneal fibrosis^26,27^. Moreover, because the NLRP3 inflammasome is thought to play a role mainly in inflammatory cells^10,15^, we assessed MGO-induced peritoneal fibrosis in myeloid cell-specific ASC-deficient mice. Unexpectedly, however, myeloid cell-specific ASC deficiency failed to inhibit MGO-induced inflammatory and fibrotic responses, and subsequent peritoneal fibrosis. Supporting this, Huen *et al*.^28^ previously reported that macrophage-specific deletion of TGF-β did not prevent renal fibrosis after ischemia-reperfusion or obstructive injury. Nezu *et al*.^29^ also reported that myeloid-specific Keap1 deletion had no effect on tubular damage in ischemia-reperfusion kidneys while tubule-specific Keap1 deletion ameliorated kidney tubular damage. These reports suggest that other cell types likely contribute to the development of fibrosis. We then focused on vascular endothelial cells for the following reasons. First, we observed that ASC deficiency had inhibitory effects on endothelial cell damage, such as hemorrhagic ascites, fibrin deposition, and the expression of PAI-1. Fibrin is considered to be derived from plasma exudation from peritoneal microvessels^30^. In addition, the levels of PAI, an inhibitor of fibrinolysis, have been shown to be increased in patients with PD and correlated with the duration of treatment^31,32^. Second, recent studies have suggested that NLRP3 inflammasome-driven inflammatory response and cell death can occur in vascular endothelial cells^33^. Furthermore, endothelial damage and angiogenesis are known to be the hallmarks of PD-related peritoneal fibrosis and EPS^4,23,34^. Using human and murine primary vascular endothelial cells, we showed that MGO markedly induces cell death through ROS generation, which is prevented by ASC deficiency, suggesting that endothelial NLRP3 inflammasome contributes to the process of PD-related peritoneal fibrosis.

Several limitations of this study should be noted. First, although we showed that NLRP3 deficiency significantly attenuates MGO-induced peritoneal fibrosis, we have not examined whether deficiency of other inflammasome-forming PRRs, such as NLRP1, NLRC4, and AIM2, could inhibit peritoneal fibrosis. In this regard, we observed no significant changes of these molecules in mice after MGO treatment; therefore, we assume that NLRP1, NLRC4, and AIM2 inflammasomes are less likely to be involved in MGO-induced peritoneal fibrosis. Second, although MGO-induced peritoneal fibrosis was significantly attenuated in IL-1β-deficient mice, we could not detect substantial IL-1β production in response to MGO in cultured endothelial cells (data now shown). Third, although we clearly showed that MGO induced ASC-dependent cell death in endothelial cells, its mechanism remains to be elucidated. Recent investigations have indicated that the inflammasome activation induces a particular form of cell death called pyroptosis by the cleavage of gasdermin D^35,36^. On the other hand, ASC has shown to be involved in other types of cell death, such as caspase-8-mediated apoptosis or necrosis distinct from necroptosis^37–40^. Thus, further studies are needed to elucidate the precise mechanism and role of NLRP3 inflammasome in PD-related peritoneal fibrosis.

In conclusion, the present study demonstrates that NLRP3 inflammasome triggers vascular endothelial cell damage and subsequent peritoneal inflammatory and fibrotic responses, resulting in MGO-induced peritoneal fibrosis. These findings identify NLRP3 inflammasome as a potential novel target for preventing and treating PD-related peritoneal fibrosis and provide new insights into the mechanism underlying this disorder.

## Materials and Methods

### Animal protocols

All animal experiments were approved by the Use and Care of Experimental Animals Committee of the Jichi Medical University (permit number 17151), and carried out in accordance with Jichi Medical University guidelines. NLRP3^–/–^, ASC^–/–^, and IL-1β^–/–^ mice were kindly provided by Drs. Vishava M. Dixit (Genentech, South San Francisco, CA), Shun’ichiro Taniguchi (Shinshu University, Matsumoto, Japan), and Yoichiro Iwakura (Tokyo University of Science, Chiba, Japan), respectively^12,41–44^. WT mice obtained from Japan SLC, Inc. (Tokyo, Japan) or ASC^+/+^ littermates were used. A similar degree of MGO-induced peritoneal thickness was observed in WT mice and ASC^+/+^ littermates. ASC^f/f^ mice were generated by homologous recombination at Phoenix Bio Co. (Utsunomiya, Japan) and crossed with LysM^cre/+^ mice (Jackson Laboratories, Bar Harbor, ME). The genetic background of all mice was C57BL/6J and 8- to 10-week-old female mice were used. Mice were housed (4/cage, RAIR HD ventilated Micro-Isolator Animal Housing Systems, Lab Products, Seaford, DE) in an environment maintained at 23 ± 2 °C with *ad libitum* access to food and water under a 12-h light and dark cycle with lights on from 8:00 to 20:00. To induce peritoneal fibrosis, mice were injected with peritoneal dialysis fluid (PDF) (100 mL/kg) containing 40 mM MGO solution (Sigma-Aldrich, St. Louis, MO) for a total of 3 weeks, 5 consecutive days per week, as described previously^7,8^. PDF contained 2.5% glucose, 100 mM NaCl, 35 mM sodium lactate, 2 mM CaCl_2_ and 0.7 mM MgCl_2_ (Midperiq 250; Terumo, Tokyo, Japan). Vehicle mice were injected with the same dosage of PDF.

### Assessment of mesentery

After the mice were euthanized and peritoneal samples were collected, the intestine was put aside by a cotton swab and cut by forceps at the site of the superior mesenteric artery. The intestine was then washed with phosphate-buffered saline (PBS), and the mesentery was extended as much as possible by cotton swabs. All of the procedures were done carefully so as not to damage the tissue. Images of the mesentery were obtained, and the area of mesentery was quantified by Adobe Photoshop CS software (ver. 8, Adobe Systems Inc., San Diego, CA).

### Histology and immunohistochemistry

The peritoneum was fixed with 4% paraformaldehyde and embedded in paraffin. The tissue sections (3-μm thick) were stained with hematoxylin and eosin (HE) and MT. For evaluation of the degree of peritoneal thickening, the thickness of the submesothelial compact zone (membrane area extending from the lower limit of the mesothelial layer to the upper limit of the muscle layer) was measured in 5 different fields of view, five measurements per field, total 25 points, at ×400 magnification using a microscope (FSX-100, Olympus, Tokyo, Japan). The mean thickness of the submesothelial compact zone in each mouse was calculated and defined as the peritoneal thickness. Immunohistochemical analyses were performed to examine the white blood cell marker CD45, the macrophage marker F4/80, the neutrophil marker Ly6G, the lymphocyte marker CD3, the endothelial marker CD34, and ASC. Briefly, deparaffinized sections were boiled in Target Retrieval Solution (Dako, Agilent Pathology Solutions, Santa Clara, CA), blocked with normal goat serum and 1% bovine serum albumin (BSA) in PBS, and incubated overnight with antibodies against CD45 (BD Biosciences, Franklin Lakes, NJ), F4/80 (Abcam, Cambridge, UK), Ly6G (Biolegend, San Diego, CA), CD3 (eBioscience, San Diego, CA), and ASC^45^. This was followed by incubation with Histofine Simple Stain Rat MAX PO (Nichirei Corporation, Tokyo, Japan). The immune complexes were detected using a DAB substrate kit (Vector Laboratories, Burlingame, CA). The sections were counterstained with hematoxylin. For immunofluorescence staining for F4/80 and ASC, the sections were incubated with primary antibodies followed by incubation with Alexa 488- or Alexa 594-conjugated secondary antibodies (Thermo Fisher Scientific, Waltham, MA) and nuclei were co-stained with 4’, 6-diamidino-2-phenylindole (DAPI; Wako Chemicals, Osaka, Japan). For immunofluorescence staining for CD34 and ASC, double labeling of two rabbit primary antibodies were performed by blocking with unconjugated Fab fragments as described previously^46^. The sections were first incubated with ani-CD34 antibody (Abcam) followed by incubation with Biotin-conjugated anti-rabbit antibody (Vector Laboratories) and Dylight Streptavidin 488 (Vector Laboratories). The sections were then blocked with 10% normal rabbit serum followed by incubation with AffiniPure Fab Fragment donkey anti-rabbit IgG (40 μg/mL, Jackson ImmunoResearch, West Grove, PA). This was followed by incubation with primary antibodies against ASC and Alexa 548-conjugated secondary antibodies. No signals were detected when an irrelevant IgG (Vector Laboratories) was used instead of the primary antibody as a negative control. The images of the stained sections were digitized and analyzed using a microscope (FSX-100) or confocal laser-scanning microscope (FV-10i, Olympus).

### Real-time RT-PCR analysis

Real-time RT-PCR analysis was performed as described previously^17,47,48^. Total RNA was prepared from the parietal and visceral peritoneum using ISOGEN (Nippon Gene Co., Ltd., Toyama, Japan) according to the manufacturer’s instructions. Real-time RT-PCR analysis was performed using the Thermal Cycler Dice Real Time Systems II (Takara Bio Inc., Shiga, Japan) to detect mRNA expression. The primers are shown in Supplementary Table S1. The expression levels of each target gene were normalized by subtracting the corresponding glyceraldehyde-3- phosphate dehydrogenase (GAPDH) cycle threshold (C_T_) values; this was done by using the ΔΔC_T_ comparative method.

### Analysis of peritoneal effluents

The peritoneal cavity was washed with 2 mL saline after the mice were euthanized. RBC counts in the peritoneal effluents were measured by an automated hematology analyzer (Celltac α, Nihon Kohden, Tokyo, Japan).

### Western blot analysis

Western blot analysis was performed as described previously^17,47,48^. Briefly, cell lysates were prepared using RIPA buffer (20 mM Tris, 2.5 mM EDTA, 1% Triton X, 10% glycerol, 1% deoxycholic acid, 0.1% SDS, 50 mM NaF, and 10 mM Na_4_P_2_O_7_·10H_2_O), and subjected to SDS–PAGE. The proteins were electrophoretically transferred to PVDF membranes. The membranes were blocked with 5% skim milk in TBS-T for 2 h at room temperature and then incubated overnight at 4 °C with primary antibodies, followed by incubation for 1 h with secondary antibodies conjugated to horseradish peroxidase (HRP). Immunoreactive bands were visualized using a Western BLoT Ultra Sensitive HRP Substrate (Takara Bio Inc.). The expression level of β-actin served as an internal control for protein loading. Primary antibodies against ASC^45^, VE-cadherin (Abcam, Cambridge. MA), and β-actin (Sigma-Aldrich) were used. HRP-goat anti-rabbit IgG (Cell Signaling Technology Inc., Boston, MA) and HRP-goat anti-mouse IgG (Cell Signaling) were used as secondary antibodies.

### Cell culture

Murine primary bone marrow-derived macrophages (BMDMs) were generated as described previously^14^. Briefly, bone marrow cells were isolated from mice and cultured in RPMI-1640 medium (Sigma-Aldrich) supplemented with 10% fetal calf serum (FCS) and 15% conditioned medium of L929 cells (ATCC, Rockville, MD) for 7 days. Primary MLVECs were isolated from the lungs of mice (8–10 weeks old, male) by modifications of a previously described protocol^49^. After the lungs were perfused with PBS via the right ventricle to remove RBCs, lung single-cell suspensions were prepared using a Lung Dissociation Kit (Miltenyi Biotec, Bergisch Gladbach, Germany). MLVECs (CD45^–^CD31^+^) were isolated by negative selection with anti-CD45-conjugated magnetic beads and positive selection with anti-CD31-conjugated magnetic beads. MLVECs were cultured on a gelatin-coated dish in 20% FCS/DMEM (Thermo Fisher Scientific), 50 μg/mL endothelial cell growth supplement (Sigma-Aldrich), 50 μg/mL heparin (Sigma-Aldrich), 5 μM SB431542 (R&D Systems, Minneapolis, MN), and 1% antibiotics at 37 °C with 5% CO_2_ for 72–96 h. Endothelial cells were identified by a cobblestone-like morphology and specific expression for VE-cadherin. MLVECs with 3–4 passages were used. HUVECs were obtained commercially (ScienCell Research Laboratories, San Diego, CA) and cultured in EC medium (ScienCell Research Laboratories). HUVECs with 5–7 passages were used for the experiments.

### Cell death assay and ROS detection

Cell death was assessed with SYTOX Green (Thermo Fisher Scientific), a membrane-impermeable DNA dye that enters dead cells. ROS generation was assessed in living cells using DCFDA (Abcam). Nuclei were co-stained with Hoechst33342. Fluorescence intensity was measured by using a multimode microplate reader (Spark TECAN, Switzerland).

### IL-1β assay

IL-1β levels were assessed using a mouse ELISA kit (R&D Systems, Minneapolis, USA) according to the manufacturer’s instructions.

### Statistical analysis

Data are expressed as the mean ± standard error of the mean (SEM). An unpaired *t* test was used to compare 2 groups. For comparisons between multiple groups, the significance of differences was determined by one-way analysis of variance (ANOVA) combined with the Tukey–Kramer test. Nonparametric data were analyzed using the Mann-Whitney U test or the Kruskal-Wallis test as appropriate. All analyses were performed using GraphPad Prism Software (ver. 7, San Diego, CA). A *p*-value of <0.05 was considered statistically significant.

## Supplementary information

Supplementary text and figures
