# Supplementary material for: Crucial Role of NLRP3 Inflammasome in the Development of Peritoneal Dialysis-related Peritoneal Fibrosis
Source: Sci Rep. 2019 Jul 17;9:10363. doi: 10.1038/s41598-019-46504-1 (PMC6637185; doi:10.1038/s41598-019-46504-1)
Supplement: Supplementary file 1 — Supplementary text and figures [file 41598_2019_46504_MOESM1_ESM.pdf]

Crucial Role of NLRP3 Inflammasome in the Development of Peritoneal Dialysis-related Peritoneal Fibrosis (Erika Hishida; Homare Ito; Takanori Komada; Tadayoshi Karasawa; Hiroaki Kimura; Sachiko Watanabe; Ryo Kamata; Emi Aizawa; Tadashi Kasahara; Yoshiyuki Morishita; Tetsu Akimoto; Daisuke Nagata; Masafumi Takahashi, SREP-1-09410-R1)

Supplementary figure legend

Supplementary Fig. S1. Expression of other inflammasome-related molecules in the peritoneum

WT mice were injected with vehicle or MGO for 21 days. mRNA expression of NLRP1, NLRC4, and AIM2 in the parietal peritoneum was assessed by using real-time RT-PCR analysis (n = 3–5 for each).

Supplementary Fig. S2. Expression of inflammatory and fibrotic markers in the visceral peritoneum

WT and $ASC^{-/-}$ mice were injected with vehicle or MGO for 21 days. (a) mRNA expression of IL-1β, IL-6, MCP-1 in the parietal peritoneum was assessed by using real-time RT-PCR analysis (n = 3–8 for each). (b) mRNA expression of collagen type 1, fibronectin, and TGF-β in the parietal peritoneum was assessed (n = 3–8 for each). Data are expressed as means ± SEM. *$p < 0.05$, **$p < 0.01$, ***$p < 0.001$.

Supplementary Fig. S3. Chylous ascites induced by MGO injection in BMT models

(a–d) $BMT^{WT\ to\ WT}$, $BMT^{ASC-/-\ to\ WT}$, $BMT^{WT\ to\ ASC-/-}$ mice were injected with vehicle or MGO for 21 days. (a) Representative images of peritoneal effluents in these BMT mice. (b) The levels of triglyceride in the peritoneal effluents. (c) Representative images of MT staining in the parietal peritoneum. (d) Quantitative analysis of peritoneal thickness (n = 5–7 for each).

Supplementary Fig. S4. Generation of $ASC^{f/f}$;$LysM^{Cre+}$ mice and IL-1β production in BMDMs

(a) Schematic diagram of wild-type ($ASC^{w/w}$), floxed-ASC ($ASC^{f/f}$), and knockout alleles after Cre-mediated excision of exon 1 to 3 ($ASC^{f/f}$; $LysM^{Cre+}$). Coding regions are highlighted as black boxes. Triangles represent loxP sites. (b) BMDMs from $ASC^{f/f}$;$LysM^{cre/–}$ and $ASC^{f/f}$;$LysM^{cre/+}$ mice were primed with 100 ng/mL LPS for 6 h and then stimulated with 2.5 and 5 μM nigericin for 1 h. The levels of IL-1β in the supernatants were assessed (n = 4 for each). Data are expressed as means ± SEM. *p<0.05, ***p < 0.001.

Supplementary table 1

Supplementary Table S1. Details of the primers used in the RT-PCR analysis

Name	5' -3' sequence	
	Forward primer	Reverse primer
NLRP3	CGAGACCTCTGGGAAAAAGCT	GCATACCATAGAGGAATGTGATGTACA
ASC	GCTGAGCAGCTGCAAACGAC	ACTTCTGTGACCCTGGCAATGAG
Caspase-1	GATGGCACATTTCCAGGACTGA	TGTTGCAGATAATGAGGGCAAGAC
IL-1β	TGAAGTTGACGGACCCCAAA	TGATGTGCTGCTGTGAGATT
TGF-β	GCAACATGTGGAACTCTACCAGA	GACGTCAAAAGACAGCCACTCA
NLRP1	TTTATCTTGGTTCCCGCTATATCG	CGGTAGCACAGCTCTAGTTCCTTT
AIM2	TTGTATCTAGGCTGATCCTGGGAC	ACCTGCACTTTGAATCAGGTGGTC
NLRC4	ATCGTCATCACCGTGTGGAG	GCCAGACTCGCCTTCAATCA
TNF-α	CCCCAAAGGGATGAGAAGTTC	GCTTGTCACTCGAATTTTGAGAA
IL-6	ACAACCACGGCCTTCCCTACTT	CACGATTTCCCAGAGAACATGTG
MCP-1	GGCTCAGCCAGATGCAGTTAAC	GCCTACTCATTGGGATCATCTTG
F4/80	CCTGGACGAATCCTGTGAAG	GGTGGGACCACAGAGAGTTG
Collagen type 1	GAGCGGAGAGTACTGGATCG	GTTCGGGCTGATGTACCAGT
Collagen type 3	CCCAACCAGAGATCCCATT	GAAGCACAGGAGCAGGTGTAGA
TGF-β	GCAACATGTGGAACTCTACCAGA	GACGTCAAAAGACAGCCACTCA
MMP-2	GACATACATCTTTGCAGGAGACAAG	TCTGCGATGAGCTTAGGGAAA
MMP-9	CCTGGAACTCACACGACATCTTC	TGGAAACTCACACGCCAGAA
PAI-1	GACACCCTCAGCATGTTCATC	AGGGTTGCACTAAACATGTCAG
TIMP-1	GTAAGGCCTGTAGCTGTGCC	AGGTGGTCTCGTTGATTTCT
GAPDH	TGTGTCCGTCGTGGATCTGA	TTCGTGTTGAAGTCGCAGGAG

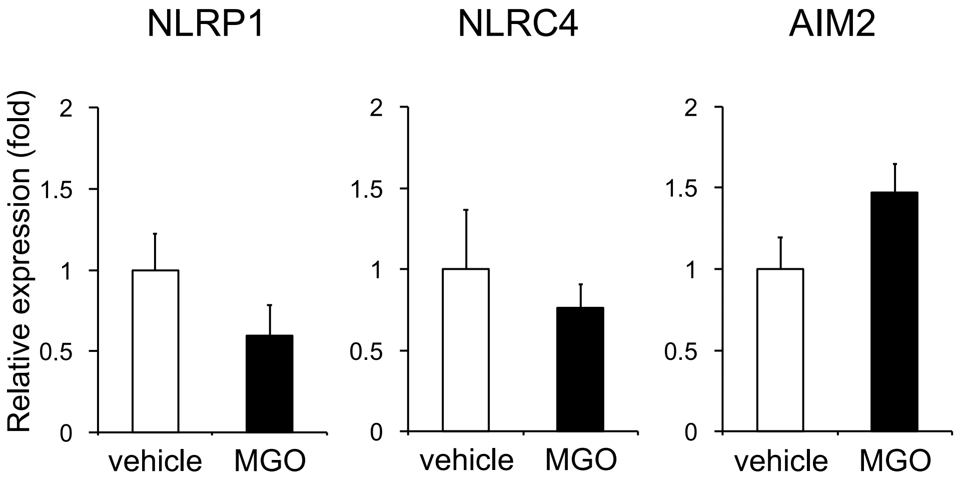
NLRP1
NLRC4
AIM2
Relative expression (fold)
2
1.5
1
0.5
0
vehicle
MGO

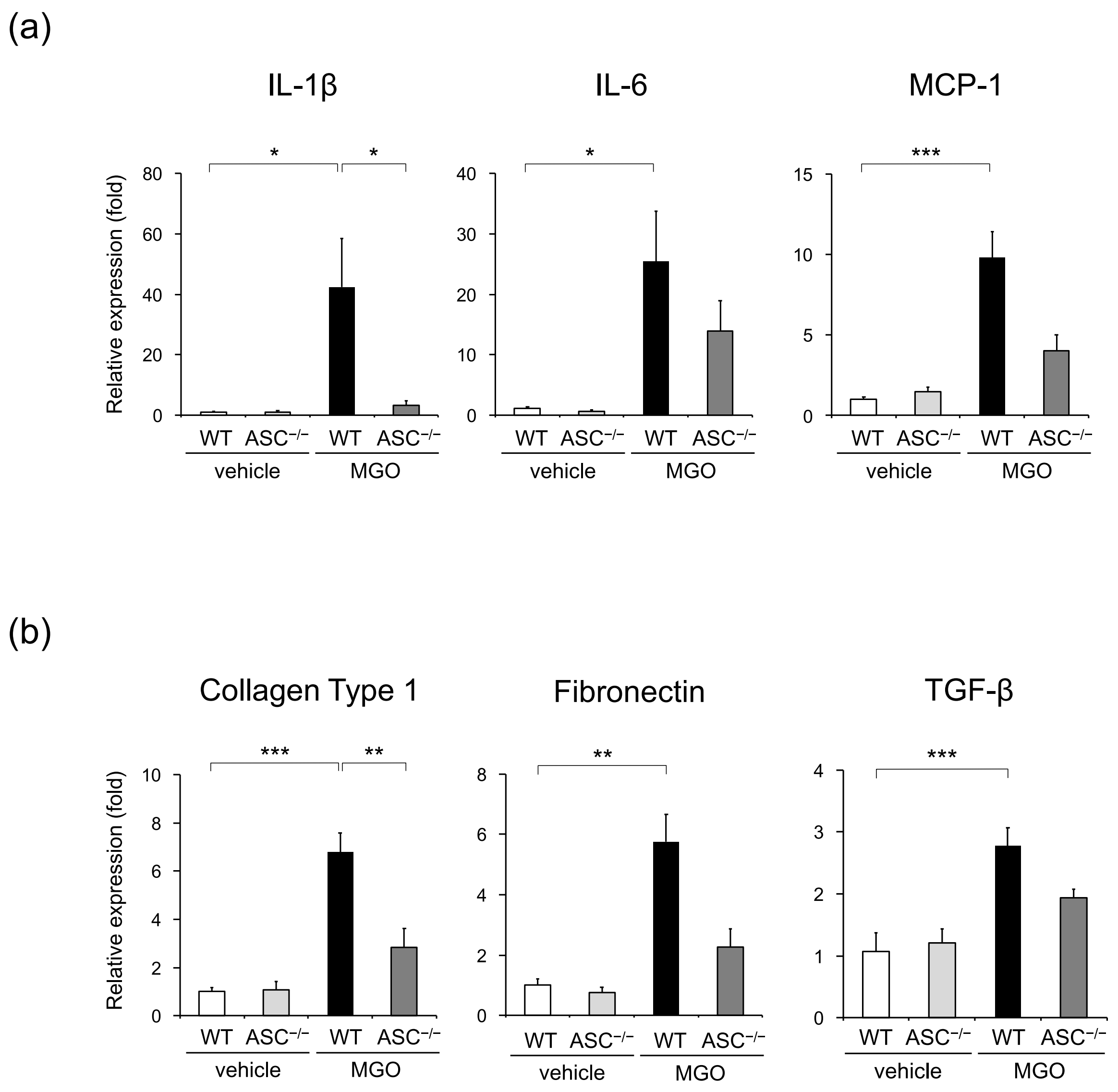
(a)
IL-1β
IL-6
MCP-1
Relative expression (fold)
80
60
40
20
0
40
30
20
10
15
10
5
*
***
WT ASC−/− WT ASC−/−
vehicle
MGO
(b)
Collagen Type 1
Fibronectin
TGF-β
***
**
10
8
6
4
2
4
3
1

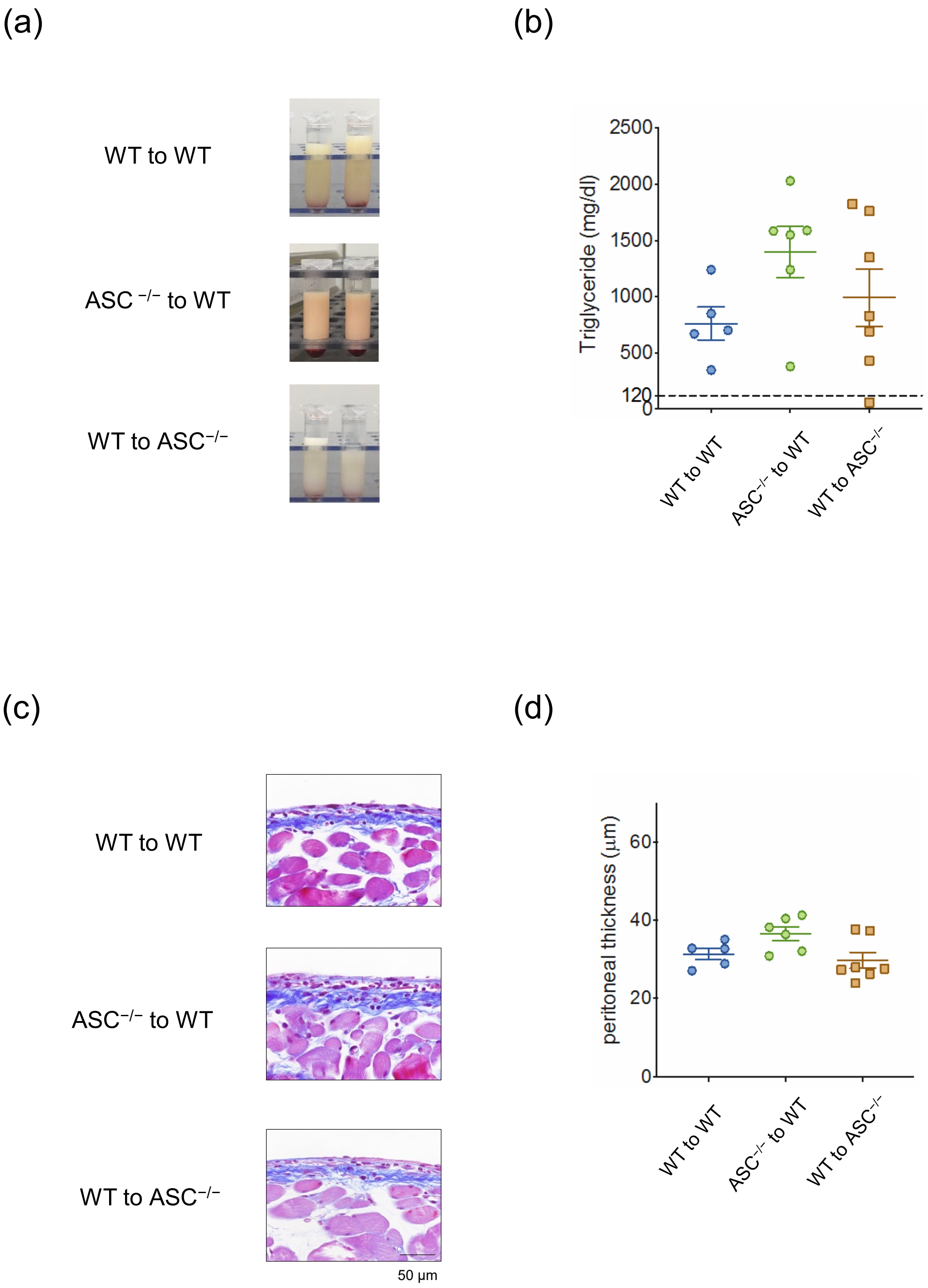
(a)
WT to WT
ASC −/− to WT
WT to ASC−/−
(b)
Triglyceride (mg/dl)
2500
2000
1500
1000
500
120
0
WT to WT
ASC−/− to WT
WT to ASC−/−
(c)
WT to WT
ASC−/− to WT
WT to ASC−/−
50 μm
(d)
peritoneal thickness (μm)
60
40
20
0
WT to WT
ASC−/− to WT
WT to ASC−/−

(a)

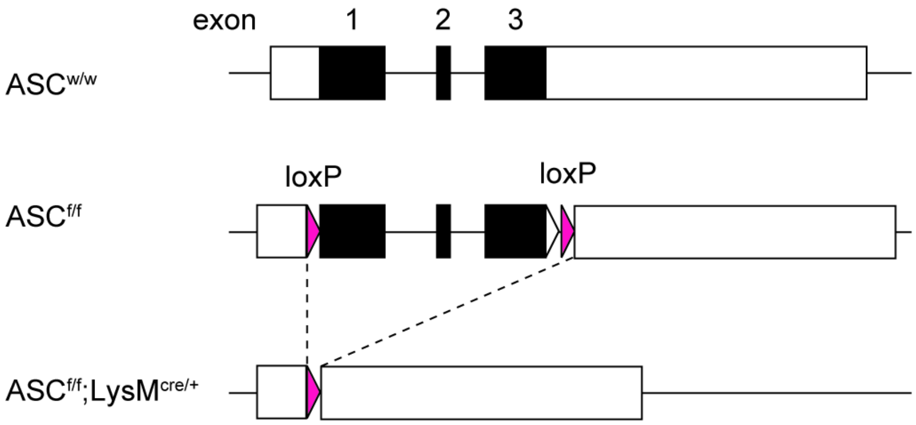
exon
1
2
3
ASC$^{w/w}$
loxP
loxP
ASC$^{f/f}$
ASC$^{f/f}$;LysM$^{cre/+}$

(b)

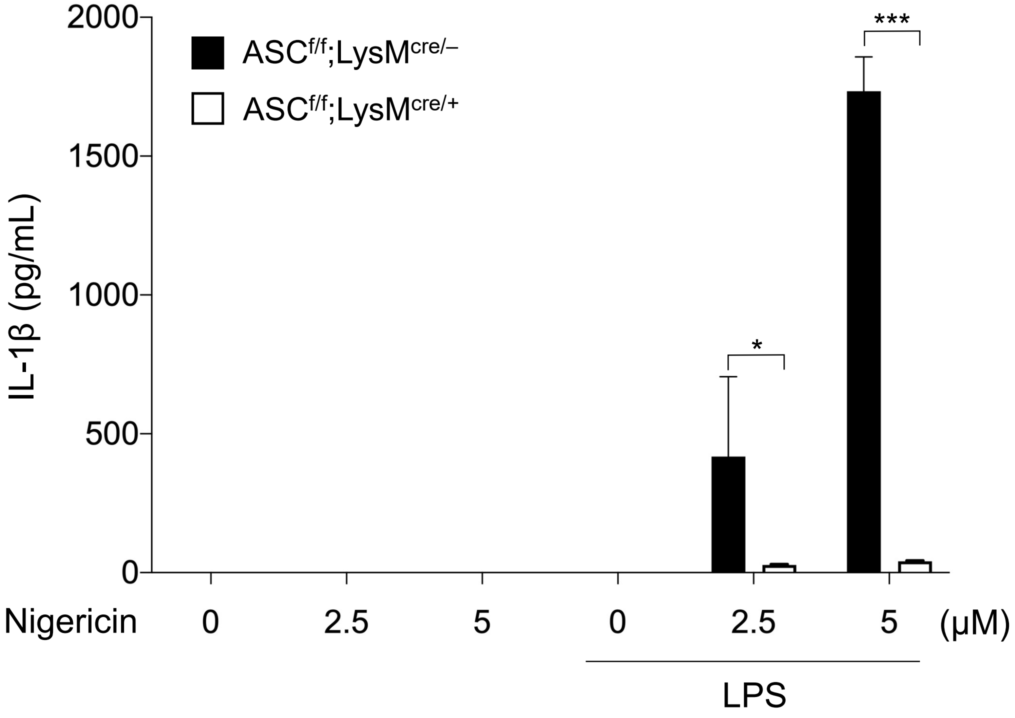
2000
1500
1000
500
0
IL-1β (pg/mL)
ASC$^{f/f}$;LysM$^{cre/–}$
ASC$^{f/f}$;LysM$^{cre/+}$
*
***
Nigericin
0
2.5
5
0
2.5
5
(μM)
LPS
